# Intracranial Electrode Location and Analysis in MNE-Python

**DOI:** 10.21105/joss.03897

**Published:** 2022-02-14

**Authors:** Alexander P. Rockhill, Eric Larson, Brittany Stedelin, Alessandra Mantovani, Ahmed M. Raslan, Alexandre Gramfort, Nicole C. Swann

**Affiliations:** 1 Department of Human Physiology, University of Oregon, Eugene OR, USA; 2 Institute for Learning and Brain Sciences, University of Washington, Seattle, WA, USA; 3 Department of Neurological Surgery, Oregon Health & Science University, Portland, Oregon; 4 Université Paris-Saclay, Inria, CEA, Palaiseau, France

## Summary

Intracranial electrophysiology analysis requires precise location and anatomical labeling of electrode recording contacts before a signal processing analysis of the data can be interpreted. Signal processing techniques are common to other electrophysiology modalities, such as magnetoencephalography (MEG) and electroencephalography (EEG), so ideally locating and labeling intracranial electrodes would be an integrated part of a single analysis software package. This work covers the addition of intracranial electrode localization and intracranial-specific analyses to MNE-Python (Gramfort et al., 2013) including aligning a computed tomography (CT) image to a magnetic resonance (MR) image, locating electrode contacts in the CT image using a graphical user interface (GUI), warping the positions of contacts from the brain of an individual to a template brain and the visualizations that accompany these steps. This allows MNE-Python’s suite of signal processing and analysis tools to be used more easily by intracranial electrophysiology researchers.

Stereoelectroencephalography (sEEG) and electrocorticography (ECoG) analyses generally require four pieces of data: the raw electrophysiology, neurosurgical planning, an MR and a CT. A preoperative MR image is collected to obtain detailed individual brain anatomy, and a postoperative CT image is collected to get a high-resolution image with 3D contact locations (although this can also be provided by a postoperative MR). The surgical plans then link the names of the contacts in the recording with their positions. This work details how to use this data to obtain the locations of contacts relative to the individual brain anatomy, the labels of the brain areas in which each contact is implanted and the locations of the contacts warped to a template brain.

To integrate these electrophysiology and imaging data, first, the CT must be aligned to the MR in order to determine the positions of the electrode contacts in the CT relative to brain structures in the MR. In MNE-Python, these are aligned using a mutual information histogrammatching approach using DIPY (Garyfallidis et al., 2014) that generally works without manual pre-alignment using the functions mne.transforms.compute_volume_registration and mne.transforms.apply_volume_registration. In the rare instances where the alignment algorithm fails, seeding it with a manual pre-alignment is supported, allowing for algorithmic precision in all cases.

Next, electrode contact locations are selected in merged CT-MR coordinates using a GUI implemented in PyQt (mne.gui.locate_ieeg). In the GUI, users click or scroll through slices to find the location of contacts. When the user navigates to the location of a channel, the channel name can be selected from a menu listing the names extracted from the recording file. Then, the user can mark the channel as associated with that location. The channel is then rendered in the 3D view as well as colored on the slice plots.

**Figure 4:**
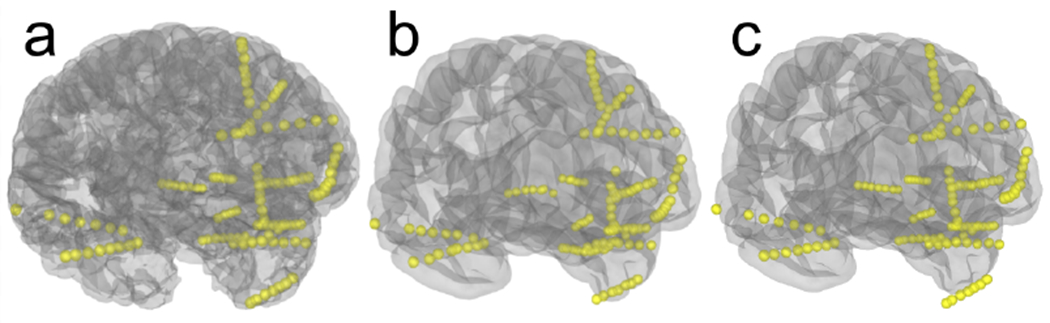
a) Example sEEG electrodes in a 3D rendering of individual subject anatomy. b) The electrodes mapped to the Freesurfer fsaverage brain using the SDR morph. c) The electrodes mapped to the Freesurfer fsaverage brain using the Talairach transform. The linear transformation in c is less accurate than the SDR transform in b; this can be seen by the electrode in the temporal pole floating outside the pial surface in c.

Located electrode contacts in merged CT-MR coordinates for an individual subject can be morphed to a template brain for group analysis using mne.transforms.compute_volume_ registration and mne.transforms.apply_volume_registration. This is done using a symmetric diffeomorphic registration (SDR; Garyfallidis et al., 2014). This non-linear mapping tends to be more accurate than a linear transformation such as the Talairach transform (Dale et al., 1999), as is shown in Figure 4.

For ECoG electrodes, “brain shift,” caused by changes in pressure during the craniotomy, can also be accounted for using MNE-Python. This shift causes grid and strip electrodes to be deeper in the post-operative CT than they were in the preoperative MR. This makes it so that the electrodes are inside the pial surface in the preoperative MRI. In MNE-Python, using mne.preprocessing.ieeg.project_sensors_onto_brain, this is compensated for by projecting the grid to the leptomeningeal surface.

**Figure 6:**
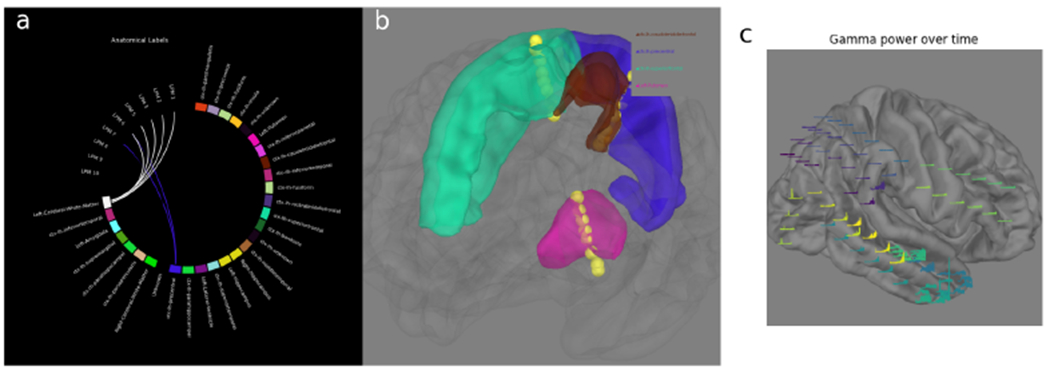
a) The anatomical labels for an sEEG electrode shaft are shown as the contacts progress from deep (starting with 1) to superficial regions. b) The anatomical surfaces that sEEG electrodes pass through are rendered along with the trajectory of the electrode shaft. c) A time-frequency decomposition of the ECoG data rendered on top of a 3D image of the brain showing the location of the grid implant.

Once the electrode locations are found both in relation to individual subject anatomy and in relation to a template brain, there are several visualization functions specific to sEEG and ECoG in MNE-Python. For sEEG, plotting the anatomical labels that the electrode shaft passes through indicates which areas are being recorded from using mne.viz.plot_channel_labels_circle (Figure 6a). These areas can also be rendered in different colors in 3D for precise visualization of the trajectory and location of the electrodes using mne.viz.Brain.add_volume_labels (Figure 6b). For ECoG, viewing a time series superimposed on a view of the 3D rendering enables the data to be displayed in relation to nearby channels using mne.viz.snapshot_brain_montage or mne.stc_near_sensors (Figure 6c).

These intracranial location and analysis steps are shown in the MNE-Python tutorials Locating intracranial electrode contacts, Working with sEEG data and Working with ECoG data, which require the dependencies nibabel (Brett et al., 2020), nilearn (Abraham et al., 2014), dipy (Garyfallidis et al., 2014), mne-bids (Appelhoff et al., 2019), pyvistaqt (Sullivan & Kaszynski, 2019) and Freesurfer (Dale et al., 1999).

## Statement of need

Integrating intracranial electrode location and analysis into MNE-Python allows researchers to go from raw data to visualizations of results using an all-in-one package that follows modern coding best practices, including unit tests, continuous integration, and thorough documentation. The integration of intracranial-specifc routines into MNE-Python provides immediate access to a wide variety of algorithms for different analyses of this data.

Previous work on intracranial software has typically come in the form of standalone packages (Groppe et al., 2017; Hamilton et al., 2017). However, these are difficult to maintain as the group of developers and number of users tends to be relatively smaller. This hampers software maintenance as the developers transition to other projects. MNE-Python is a generalpurpose electrophysiology analysis package which makes it easier to retain a larger core group of developers and makes it more likely that this package and the intracranial functionality will be maintained and improved on in the long-term. MNE-Python also has stability due to the funding it receives directly for development from institutions such as National Institutes of Health, the Chan Zuckerberg open-source initiative, the European Research Council and Agence Nationale de la Recherche in France.

Other general purpose packages, written in MATLAB, provide similar functionality (Oostenveld et al., 2011; Tadel et al., 2011) but can be difficult for researchers to integrate into Python-based analyses and require a MATLAB license. Compared to alternatives, MNEPython has extensive unit tests and continuous integration across Windows, Mac OS and Ubuntu operating systems, which helps ensure the stability of the code base. Previous work in Python also provides similar functionality (Hamilton et al., 2017) but was not designed for sEEG electrode location. Adding the ability to control various aspects of the visualization (e.g., electrode contact marker size, opacity, multiple views) makes this task much easier, both for sEEG and ECoG. Additionally, compared to this previous work, MNE-Python has added key features. First it utilizes an SDR morph to map electrode contact locations onto a template brain, which is orders of magnitude faster (approximately 15 minutes compared to 15 hours) and is comparably accurate (Avants et al., 2008). Second, it has a “snap to center” of the electrode contact feature to both increase accuracy and repeatability. This feature uses the center of mass of all voxels that monotonically decrease from the highest intensity voxel nearby the selected location. Next, it has an integrated 3D view which updates upon selection. Finally, it uses a predetermined list of channel names from the recording file to speed up the localization process and to eliminate matching errors. In general, the intracranial electrode location and analysis in MNE-Python is implemented using coding best-practices, it is feature complete for intracranial analysis, and it has user-friendly features. Importantly, it is poised to be a well-maintained tool into the future.

## Figures and Tables

**Figure 1:**
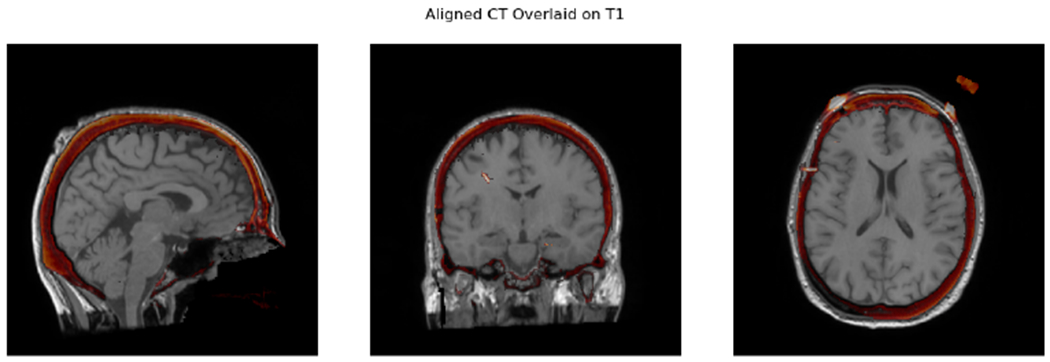
A CT image correctly aligned to the MR image. The darker areas in the MR are the skull whereas the lighter, outermost regions are subcutaneous fat. The CT is shown with a red scale, and red areas shown in the slice are the skull, which has high intensity in the CT.

**Figure 2:**
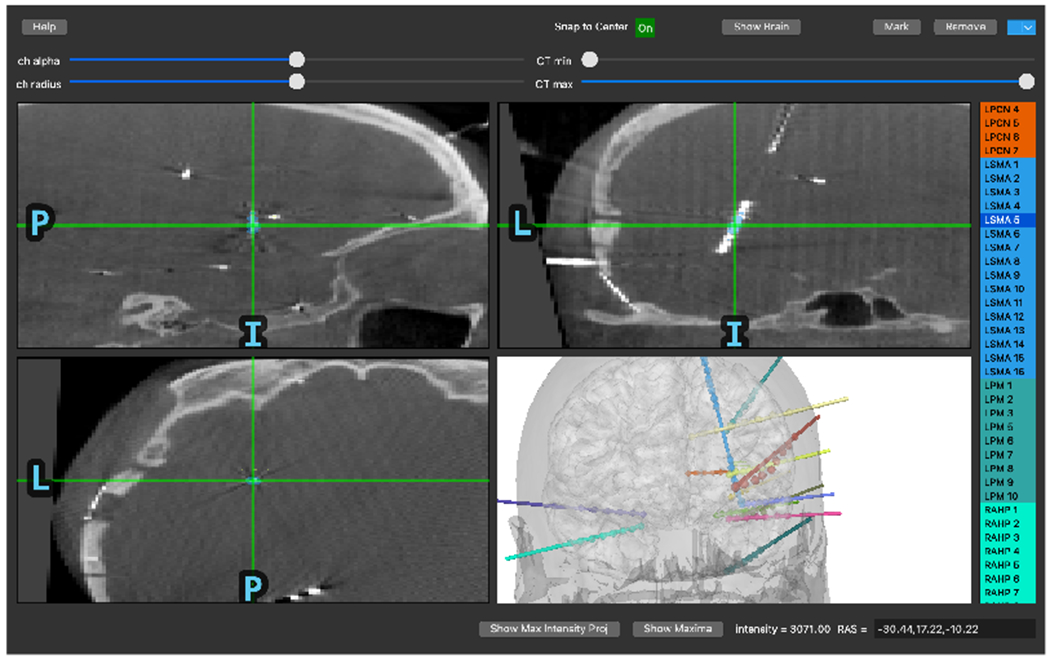
A screenshot of the GUI during a sEEG electrode location. For sEEG, the 3D brain view is typically similar to the surgical plans, making it easier to determine which electrode is which. With only 2D slice plots, locations and directions must be pieced together, which is substantially more difficult.

**Figure 3:**
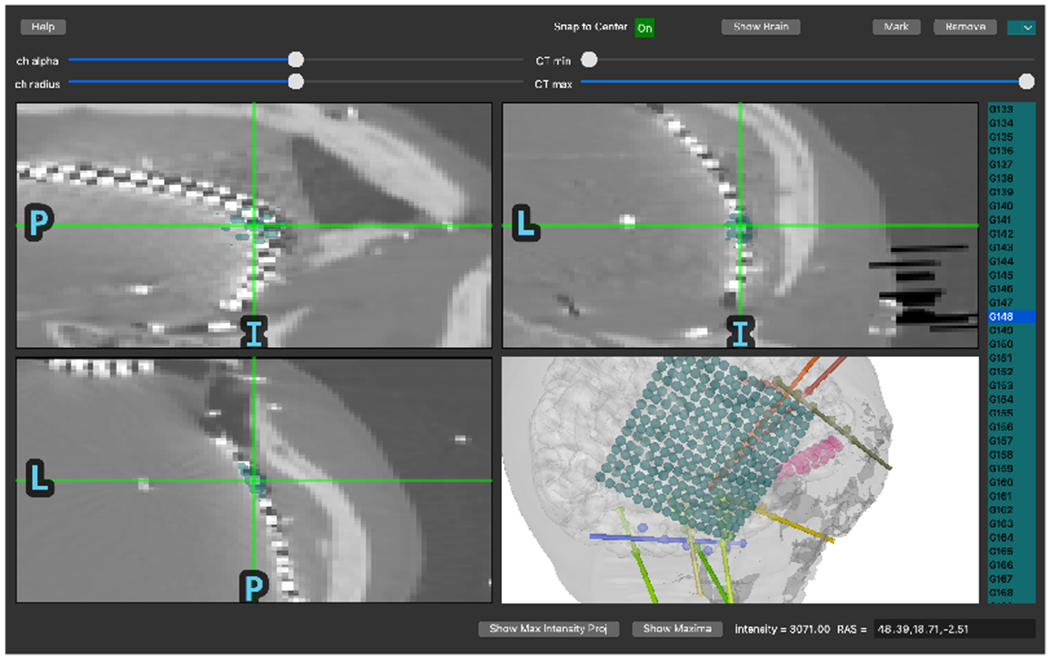
A screenshot of the GUI during an ECoG electrode location. For ECoG, the 3D brain view makes it easier to select each contact in a large grid. Without the 3D view, it is difficult to continue in one row without jumping to another row incorrectly when viewing in 2D. These mistakes make it much slower to identify contacts.

**Figure 5:**
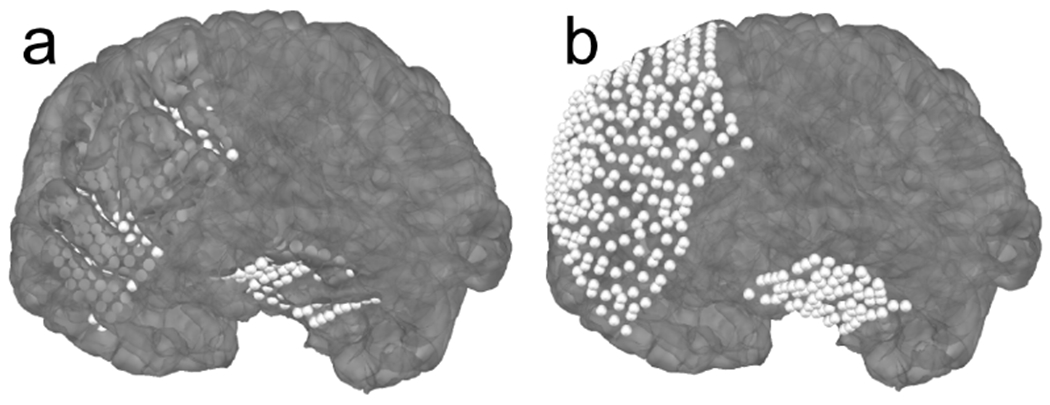
a) A 3D rendering of an ECoG grid without correction for “brain shift.” In this case, the electrode contacts are under the pial surface of the preoperative brain because the change in pressure shifted the brain during the operation. b) The same 3D rendering with the “brain shift” correction.
